# CAR‐T therapy: Prospects in targeting cancer stem cells

**DOI:** 10.1111/jcmm.16939

**Published:** 2021-09-28

**Authors:** Xiaoyue Cui, Rui Liu, Lian Duan, Dan Cao, Qiaoling Zhang, Aijie Zhang

**Affiliations:** ^1^ Basic Laboratory Suining Central Hospital Suining China; ^2^ Department of Breast and Thyroid Surgery Suining Central Hospital Suining China; ^3^ Key Laboratory of Metabolic Diseases Suining Central Hospital Suining China

**Keywords:** cancer stem cells, CAR‐T, immunotherapy, targeted therapy

## Abstract

Cancer stem cells (CSCs), a group of tumour cells with stem cell characteristics, have the ability of self‐renewal, multi‐lineage differentiation and tumour formation. Since CSCs are resistant to conventional radiotherapy and chemotherapy, their existence may be one of the root causes of cancer treatment failure and tumour progression. The elimination of CSCs may be effective for eventual tumour eradication. Because of the good therapeutic effects without major histocompatibility complex (MHC) restriction and the unique characteristics of CSCs, chimeric antigen receptor T‐cell (CAR‐T) therapy is expected to be an important method to eliminate CSCs. In this review, we have discussed the feasibility of CSCs‐targeted CAR‐T therapy for cancer treatment, summarized current research and clinical trials of targeting CSCs with CAR‐T cells and forecasted the challenges and future direction from the perspectives of toxicity, persistence and potency, trafficking, infiltration, immunosuppressive tumour microenvironment, and tumour heterogeneity.

## 1 INTRODUCTION

The hypothesis that tumours originated from “stem cells” was first proposed about 150 years ago. However, relevant research had been progressing slowly until a growing number of experimental data strongly supported the tumour stem cell hypothesis which has changed views on tumourigenesis and tumour cell biology.^1^, ^2^ In cancer stem cell theory, tumours originate from a small portion of cancer stem cells (CSCs), and they have the capacity of immortal proliferation and multi‐lineage differentiation, which drives tumour formation, growth, recurrence, metastasis, drug resistance, chemo/radio‐resistance and other malignant phenotypic characteristics.^3^ In this context, while CSCs are resistant to radiotherapy, chemotherapy and certain targeted therapies, the key to cancer treatment remains in CSCs. In addition, because of the special tumour‐killing mechanism, cancer stem cell may be more sensitive to immunotherapy, and in‐depth study of CSCs characteristics may also significantly promote the development of tumour immunotherapy.^4^

Recently, the clinical application of chimeric antigen receptor T (CAR‐T)‐cell therapy has made an unprecedented breakthrough in the treatment of haematological diseases.^5^ The safety and feasibility of CAR‐T therapy in the treatment of solid tumours have also been confirmed.^6^ For CAR‐T therapy, T cells from the patients will be genetically engineered to express chimeric antigen receptors (CAR), and then be adoptively transferred back to patients. The genetically engineered CAR‐T cells will recognize the surface antigen of tumour cells and selectively target and kill those tumour cells.^7^ CAR‐T cells can recognize the target antigen independently of MHC restrictions.^8^ After the recognition, CAR‐T cells fix their position specifically in the tumour site and can have sustained persistence for a while. The successful application of CAR‐T cells in cancer treatment represents a milestone in anticancer therapy.

Cancer stem cells have some unique characteristics, such as slow rate of division, high expression of drug efflux pumps,^9^ heightened activation of DNA repair mechanisms^10^ and microenvironment characteristics: hypoxia and acidosis,^11^ which is due to the expression of specific surface markers. These surface markers can be used as specific targets for CAR‐T therapy to eliminate CSCs. In addition, the expression of MHC molecules on the surface of CSCs is low, which causes MHC restriction when immunotherapy is used to target CSCs.^8^ However, in CAR‐T therapy, CAR‐T cells can recognize the target antigen with no MHC restrictions,^8^ which endows some advantages for the application of CAR‐T therapy to eliminate CSCs. In this review, we analysed the feasibility of targeting CSCs by using CAR‐T cells, summarized published studies on CSCs‐targeted CAR‐T therapy, pointed out the challenges of targeting CSCs by CAR‐T cells related to toxicity, persistence and potency, trafficking, infiltration, immunosuppressive tumour microenvironment, tumour heterogeneity and purposed promising strategies, such as novel CAR containing a JAK‐STAT signalling domain, modulation of chemokine signalling, directing CAR‐T cell to target vascular endothelial growth factor receptor 2(VEGFR2), combining CSCs‐targeted therapy with FDA‐approved PD‐1/PD‐L1 checkpoint inhibitors, multi‐target CAR‐T cell therapies and transgenic modification of the CAR structure, for the future development of CSCs‐targeted therapy.

## 2 GENERATION OF CAR‐T CELLS FOR CANCER IMMUNOTHERAPY

Chimeric antigen receptor T‐cell therapy has emerged as a novel therapeutic T‐cell engineering practice, in which T cells derived from patient blood were engineered *in vitro* to express artificial receptors to target a specific tumour antigen, then the modified T cells will be adoptively infused back to the patient's body to fight against cancer.^12^ For the preparation of CAR‐T cells, activated T cells were infected with retroviruses or lentiviruses loaded with CAR sequences to express receptors, and these modified T cells can recognize tumour‐associated antigens and express the tandem co‐stimulation molecular signal transduction fragments which were related to T‐cell activation.^13^ The engineered CAR‐T cells were expanded *in vitro* and then infused into patients to fight against tumours. In 1989, Gross et al. first proposed the concept of CAR‐T cell therapy.^14^ At present, this therapy has made breakthroughs in clinical trials for the treatment of leukaemia, and has gradually extended to the clinical treatment of solid tumours.^15^

The development of CAR‐T structure has gone through four generations. Each generation of CAR‐T structure is modified by adding more components in the intracellular space to make it more specific, efficient and durable (Table 1). The first generation of CAR‐T cells composed of the single‐chain variable fragment (scFv) and an intracellular CD3ζ signalling domain for T‐cell activation mainly solved the problem of targeting, but it lacked complete costimulatory signals and cannot fully activate T cells which limited its antitumour activity.^16^ Subsequently, second‐ and third‐generation CARs were invented, which included one or two costimulatory domains respectively. The second‐generation CAR‐T cells had one costimulatory domain – CD28 or 4‐1BB, which effectively improves the tumour‐killing effect.^17^ The third‐generation CAR‐T cells carried two costimulatory molecular domains, which significantly increased the proliferation activity of CAR‐T cells and enhanced cytokines release, which improves the *in vivo* persistence of CAR‐T cells and results in a stronger cytotoxic activity.^18^ More recently, the fourth‐generation CAR‐T cells, also called TRUCK‐T (CAR redirected T cells that deliver a transgenic product to the targeted tumour tissue) cells, were engineered to secrete specific cytokines, such as IL‐12, IL15, IL‐18, CCL19 and IL‐7, so as to overcome the suppression from the tumour immune microenvironment, recruit and activate the second wave of immune cells to produce an immune response.^19^

**TABLE 1:** Architectural evolution of CAR‐T cell design

Generation	The evolution of chimeric antigen receptors (CARs)
1st generation	First‐generation CARs contain the CD3ζ chain of the T‐cell receptor complex
2nd generation	Second‐generation synthetic antigen receptors differ from the first generation by the addition of a costimulatory domain (either CD28 or 4‐1BB).
3rd generation	Third‐generation CARs contain two costimulatory domains, respectively, such as CD28 and OX40.
4th generation	Fourth‐generation CARs, the so‐called TRUCKs or armoured CARs which are additionally modified with a constitutive or inducible expression cassette for a transgenic protein, which is released by the CAR‐T cell to modulate the T‐cell response.
Other evolution	Introducing some regulatory elements into CAR‐T cells, which include suicide‐initiated, negative‐regulatory and switch‐initiated components, or using dual antigen‐targeting CARs and inhibitory CARs.

In addition to the evolution of CAR designs outlined above, some elements with regulatory functions can be added with the expression of an “armour” protein, by introducing suicide‐initiated, negative‐regulatory and switch‐initiated components into CAR‐T cells to have better control of the cytotoxic response of engineered CAR‐T cells on tumours.^20^ In recent years, some new types of CAR‐T cells have been developed to increase the safety and therapeutic effect of CAR‐T cell therapy, such as dual antigen‐targeting CARs which improved specificity through targeting multiple antigens, and inhibitory CARs which were engineered to inhibit T‐cell activation upon binding to an antigen expressed on non‐malignant cells instead of tumour cells^21^, ^22^ (Table 1).

**FIGURE 1:**
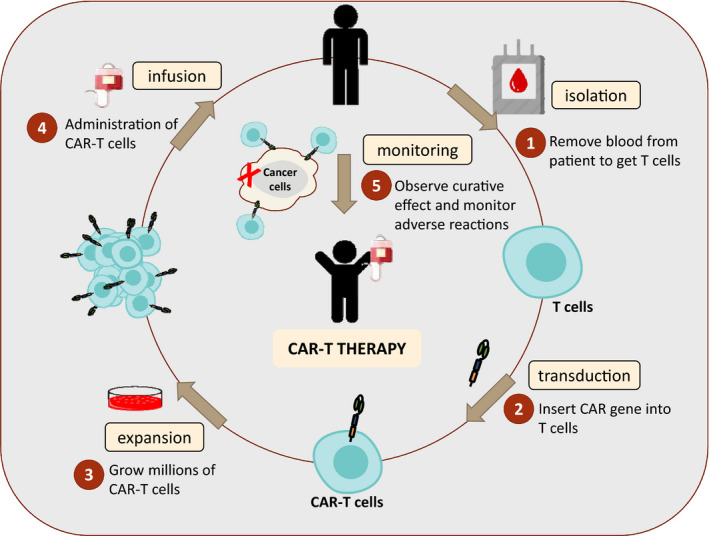
Diagram of CAR‐T treatment process. CAR‐T cell therapy can be defined as a treatment in which a patient's T cells are genetically modified in laboratory to express chimeric antigen receptors (CARs) and attack cancer cells. The process of adoptive CAR‐T cell therapy includes the following steps:(1) isolation of T cells from the peripheral blood sample by the process of leukapheresis; (2) transduction of cells by a viral vector encoding the CAR gene; (3) expansion of in vitro CAR‐T cells; (4) administration into the patient to kill cancer; (5) monitoring: observe curative effect and monitor adverse reactions

The CAR‐T cell therapy currently used in clinical practice is based on the second‐generation CAR‐T cells and mainly targets B‐cell‐related diseases, while the clinical application of the third‐ or fourth‐generation CAR‐T cells is at the early stages.^23^ Since the infusion of allogeneic T cells is prone to cause human immune rejection, CAR‐T treatment currently uses patient's own T cells. As shown in Figure 1, the general treatment process can be divided into five steps: (1) Separation: isolate and purify T cells from the peripheral blood mononuclear cell (PBMC) of tumour patients through leucapheresis; (2) Modification: genetic engineering technology is applied to prepare CAR‐T cells through adding a chimeric antibody to recognize tumour cells and activate T cells at the same time; (3) Expansion: culture CAR‐T cells *in vitro* to the required dose for treatment, generally at the level of one billion to ten billion; (4) Infusion: infuse CAR‐T cells into patients with cancer; (5) Monitoring: observe the efficacy and closely monitor the adverse reactions.^24^ The advantages of CAR‐T cell therapy include potential *in vivo* proliferation, long‐term *in vivo* persistence and efficient homing of CAR‐T cells to the tumour site.^25^ Some of these patients have achieved durable complete remissions (CRs), which standard cytotoxic chemotherapy seldom achieves.^26^ The advantages and encouraging results of CAR‐T therapy mentioned above make us optimistic about its clinical prospects.

## 3 ANALYSES OF THE FEASIBILITY AND ADVANTAGES OF TARGETING CSCS WITH CAR‐T THERAPY FOR CANCER TREATMENT

Among many anticancer therapies, the primary problem impediment against cancer curability is tumour recurrence, which is mainly caused by the presence of CSCs.^27^ Recently, cell therapy represented by CAR‐T cells has shown strong curative effects in tumour treatment and has the tendency to become an essential tumour treatment strategy.^28^ Based on the characteristics of CAR‐T cells and CSCs, we proposed that targeting CSCs with CAR‐T therapy is feasible, and analysed the advantages and feasibility of this promising therapy.

### 3.1 Boost the body's own immune system to eliminate CSCs

Cancer stem cells play an important role in tumour occurrence, drug resistance, cancer recurrence, invasion and metastasis. Therefore, the elimination of CSCs improves tumour‐killing efficacy and even achieves radically curing tumours. Current strategies of targeting CSCs mainly include: (1) Targeting surface biomarkers of CSCs, mainly by developing monoclonal antibodies that specifically target the CSCs biomarkers^29^; (2) Targeting key intracellular signal transduction pathways, such as NOTCH, Hedgehog (Hh) or WNT/ß‐Catenin signalling pathway^30^, ^31^, ^32^; (3) Inhibiting ATP‐binding cassette transporters, for example, use Verapamil, Vardenafil and Laniquidar to inhibit the ABC transporters or use RNAi for ABC transporter gene silencing^33^; (4) Induces CSCs differentiation and changes the microenvironment of CSCs.^34^ Although a variety of strategies mentioned above have been used to target CSCs, these treatments are currently only in the laboratory stage. Up to now, no effective therapies have been verified by clinical trials, and there are still no drugs specifically used for targeting CSCs in clinical application.^35^

Harnessing the power of the immune system to target CSCs is a promising therapeutic approach. For instance, J.C. Sun et al. (2010) applied dendritic cell‐based vaccines, which were treated with antigens from CD133+ hepatocellular carcinoma cells to activate specific cytotoxic lymphocytes, and therefore destroyed hepatocellular carcinoma CSCs.^36^ In the last decade, cell‐based immunotherapy represented by CAR‐T therapy is being considered as an efficient approach for the treatment of cancer.^6^ This is a way to eliminate tumour stem cells using the power of the body's own immune system, based upon the principle of targeting the surface markers of CSCs. CAR‐T cells eliminate CSCs relying on body's own immune system, and the infusion of millions of exogenous modified T cells can highly enhance the body's immune function.

### 3.2 The unique characteristics of CSCs are suitable for CAR‐T therapy

Targeting CSCs may realize tumour radical eradication, while the CSCs are protected by their unique characteristics, such as infrequent replication, enhanced drug resistance and heightened activation of DNA repair mechanisms as we mentioned in part 2. However, some of the CSCs characteristics may be harnessed to eradicate CSCs. For example, ATP‐binding cassette subfamily B member 5 (ABCB5), a marker of CSCs in a number of malignancies and a drug efflux transporter which associates multidrug resistance, tumour progression and recurrence, can be used in the tumour eradication.^37^, ^38^ Treatment with anti‐ABCB5 monoclonal antibodies has been shown to inhibit tumour growth in xeno‐transplantation models which prove that ABCB5 could be a good target for CSCs eradication.^39^ Furthermore, as ABCB5‐reactive CD8+ T cells are present in the peripheral blood of melanoma patients and an ABCB5‐specific response can be induced *in vitro* in naive donors, which implicate that ABCB5 could be a potential target for cancer immunotherapy.^40^ The subcellular location for ABCB5 expression is on cell membrane, which creates conditions for CAR‐T therapy targeting at ABCB5.^41^ Interaction between CAR and ABCB5 helps to the formation of immune synapse, through which the contact‐dependent cytotoxicity may occur.

### 3.3 CAR‐T is an MHC‐independent adoptive cellular immunotherapy

CAR‐T therapy stands out among many CSCs‐targeted therapies for it is an MHC‐independent adoptive cellular immunotherapy. In 1975, Doherty and Zinkernagel first proposed the phenomenon of “MHC restriction” – viral peptides can only be recognized by T cells when combined with specific MHC molecules.^42^ MHC is an important component of the immune system and plays a key role in antigen presentation, enabling specific T lymphocytes detect foreign antigen.^43^ However, the expression of MHC molecules on CSCs is lower, which may prevent the body from boosting immune system to eliminate CSCs.^44^ Fortunately, CAR‐T therapy is a MHC‐independent adoptive cellular immunotherapy for the unique structure of single‐chain variable fragment (scFv), mainly formed by variable regions of heavy and light chains, which can recognize cell surface antigens directly and specifically, instead of being restricted by the down‐regulation of MHC molecules.^45^ Therefore, although CSCs are not easily eliminated by the immune cells from the immune system of patients, it is feasible to eliminate CSCs through CAR‐T therapy.

### 3.4 The existence surface antigens of CSCs that can be targeted by CAR‐T cells

Chimeric antigen receptor T‐cell cells can specifically recognize the surface antigens of tumour cells and effectively inhibit their growth and proliferation, which suggests that molecular markers on CSCs may be used as targets for CAR‐T cell immunotherapy. The discovery of surface markers of CSCs provides specific therapeutic targets for the treatment of CSCs. A large number of previous experiments have identified the expression of CD133, CD90, ALDH and EpCAM in CSCs of many types of cancers.^46^ These markers can be used as an important molecular target for CAR‐T cells to kill CSCs to achieve the therapeutic effect of eliminating CSCs and inhibiting tumour recurrence and metastasis. In addition, certain molecular markers expressed in common tumour cells, such as epidermal growth factor receptor variant III (EGFRvIII), chondroitin sulphate proteoglycan 4 (CSPG4), human epidermal growth factor receptor 2(HER2) and NKG2D ligands (NKG2DLs), etc., are also expressed on the surface of tumour stem cells.^47^, ^48^, ^49^ The construction of CAR‐T cells with molecular markers of CSCs as targets has a certain theoretical effect in the elimination of CSCs.

## 4 REPORTED LABORATORY RESEARCH OF CSCS‐TARGETED CAR‐T THERAPY

It is necessary that CSCs‐targeted CAR‐T cells efficiency, cytolytic activities and CAR molecule expression must be evaluated in preclinical setting before utilizing these cells as a therapy. In the following part, after carefully investigating laboratory studies on targeting CSCs with CAR‐T therapies from very limited number of reports, we proposed that the existing laboratory research on CSCs‐targeted CAR‐T therapies can be divided into two categories (Table 2): (1) the first category is targeting specific antigen molecules of CSCs, such as CD133, EpCAM or ALDH and designing corresponding CAR‐T cells for carrying out *in vitro* killing experiments and *in vivo* animal experiments for verification and (2) the second category is targeting “general” antigens (antigens also expressed in cancer cells) on CSCs.

**TABLE 2:** Current studies on CSCs‐targeted CAR‐T therapy

Antigen	Tumour target	Type of CARs	Target cells	Animal experiment	Main findings	Specific target
CD133	Glioblastoma	3rd generation CAR	AC133+ patient‐derived NCH421k GBM‐SCs	Orthotopic NMRI mouse model of GBM	CSCs isolated from glioblastoma patients were successfully killed by anti‐CD133 CAR‐T cells both *in vitro* and *in vivo* models of orthotopic tumour.^50^	Yes
EpCAM	Human prostate cancer	2nd generation CAR	PC3M cells and PC cells	NOD/SCID mice (injected with PC3M‐luc cells)	Anti‐EpCAM CAR‐T cells were able to eliminate PC3M cells which express high levels of EpCAM *in vivo* and *in vitro*, as well as able to inhibit the tumour growth of PC3 cells that express low levels of EpCAM and prolong mouse survival in a PC3 metastasis model.^51^	Yes
EpCAM	Human ovarian and colorectal cancer	3rd generation CAR	EpCAM‐positive ovarian cancer cell lines	NSG mouse xenograft model of human ovarian and colorectal carcinoma	CAR‐T cells targeting EpCAM on human ovarian and colorectal cancer cells are capable of killing the cancer cells *in vitro*, and the adoptive transfer of these CAR‐T cells resulted in prolonged animal survival by eradicating the established ovarian xenografts^52^	Yes
HER2	Glioblastoma	2nd generation CAR	Glioblastoma stem cells	Orthotopic xenogeneic SCID mouse model of GBM	Patients' HER2‐specific CAR‐T cells killed CD133‐positive and CD133‐negative cells derived from primary HER2‐positive glioblastomas. These HER2‐specific T cells had a potent antitumor activity against autologous tumours in an orthotopic xenogeneic SCID mouse model^48^	No
EGFRvIII	Glioblastoma	2nd and 3rd generation CAR	Glioma stem cells(GSCs): GSCs (0308, 1228, and 0822)	NA	Anti‐EGFRvIII CAR‐engineered T cells produced the effector cytokine, IFN‐γ and lysed antigen expressing target cells.^49^	No
CSPG4	Glioblastoma	2nd generation CAR	Glioblastoma stem cells	NA	It is reported for the first time that CSPG4 is expressed on glioblastoma cancer stem cells (GSC) and demonstrate that anti‐CSPG4 CAR‐transduced T cells recognize and kill these GSC.^55^	No
NKG2DLs	Glioblastoma	2nd generation CAR	Patient‐derived GSC‐3#	B‐NDG mice bearing U251MG xenografts	NKG2D ligands‐targeted CAR‐T cells efficiently lysed glioblastoma cells and cancer stem cells *in vitro* and produced high levels of cytokines, perforin and granzyme B. *In vivo*, the CAR‐T cells markedly eliminated xenograft tumours and did not exhibit significant toxicity in the treated mice^47^	No

In the experiment of co‐culturing glioma stem cells with CAR‐T cells, Zhu et al. proved that anti‐CD133 CAR‐T cells can kill tumour patients‐derived glioma stem cells *in vitro* and have also shown therapeutic effects in animal models of orthotopic tumours.^50^ However, in this study, when CAR‐T cells have an indirect contact with CD57‐positive target cells, the expression of CD57, which is a marker to abrogate T‐cell function, on CAR‐T cells is rapidly up‐regulated, leading to function impairment of CAR‐T cells. In another study by Deng et al., it is proved that EpCAM‐specific CARs can inhibit the growth of the human prostate cancer cell line PC3, a kind of cell line with low expression levels of EpCAM or kill PC3M (a highly metastatic clone of PC3 with high expression of EpCAM) *in vitro* and *in vivo*. Interestingly, despite the low expression level of EpCAM on PC3 cells, lymphocytes targeting EpCAM can cause significant tumour‐killing effects and inhibit the metastasis of PC3 cells in NOD/SCID mice.^51^ For further study, Ang et al. reported that anti‐EpCAM CAR‐T cells exhibited specific *in vitro* killing activity against EpCAM‐positive human ovarian and colorectal cancer cells, and successfully treated local peritoneal cancer in xenograft mice with anti‐EpCAM CAR‐T cells, which show the feasibility of this therapy for curing clinical gastrointestinal and gynaecological malignancies.^52^

Compared with targeting the specific surface antigens, engineering the corresponding CAR‐T cells to target “general” CSCs markers is another way to eliminate CSCs with CAR‐T therapy. General antigen‐targeted CAR‐T cells may not be initially designed to specifically kill CSCs. Due to the expression of these markers was also detected on the surface of CSCs, when such CAR‐T cells were co‐cultured with corresponding CSCs, they also have a cancer‐killing effect (Table 2). HER2 is a tumour‐associated antigen that is expressed by up to 80% of glioblastomas (GBMs) but not by normal postnatal neurons or glia.^53^ Ahmed et al. generated HER2‐specific T cells from 10 consecutive glioblastoma patients with a retroviral vector encoding a HER2‐specific CAR to produce effector cells. They showed that these effector cells recognized autologous HER2‐positive GBMs including their CD133‐positive stem cells *in vitro* and had potent antitumor activity in an orthotopic, xenograft model.^48^ EGFRvIII is one of the few tumour‐specific antigens and thus an attractive candidate for the development of immunotherapy for glioblastoma patients. Richard A. Morgan et al. sought to develop immune‐based approaches targeting GCS as a potential treatment for glioblastoma, and report for the first time that EGFRvIII is expressed in glioblastoma cancer stem cells (GSC) lines and EGFRvIII CAR‐engineered T cells effectively target these lines.^49^ Moreover, CSPG4, a highly immunogenic cell surface proteoglycan identified on melanoma cells, has been shown to facilitate the progression from radial to vertical growth in melanoma tumours.^54^ Rachel E Beard et al. report for the first time that CSPG4 is also expressed on GSC and demonstrate that anti‐CSPG4 CAR‐transduced T cells recognize and kill these GSC.^55^ Similarly, the expression of NKG2DLs is usually expressed in most epithelial‐derived tumour cells, such as ovarian cancer, colon cancer and leukaemia, while it is rarely detected in healthy adult tissues.^56^ Yang et al. confirmed the high expression of NKG2DLs in GSCs and verified anti‐NKG2DLs CAR‐T cells efficiently lysed glioblastoma cells and cancer stem cells *in vitro*, and obviously eliminated xenograft tumours and did not exhibit significant toxicity in the mice model.^47^

The above experimental results indicated that adoptive cellular immunotherapy with CSCs‐targeted CAR‐T cells is expected to become a promising cancer treatment method. For other common markers of CSCs, such as CD90 and ALDH that could be theoretically ideal targets for CAR‐T therapy, unfortunately, there is no reported studies using CD90 or ALDH‐specific CAR‐T cell for cancer treatment.

## 5 CLINICAL APPLICATION OF CSCS‐TARGETED CAR‐T THERAPY

To investigate the clinical application of CSCs‐targeted CAR‐T therapy, we have searched the ClinicalTrials.gov website and summarized the latest registered clinical trials of CAR‐T therapy using surface markers of CSCs (Table 3). These trials are within Phase I or Phase II, most of which are carried out in China and performed in recruiting stage. Twenty‐one trials are specific to haematological tumours treatment, in which acute myelocytic leukaemia (AML) treatment accounts for the majority; seven trials are for treating solid tumours, such as nasopharyngeal carcinoma (NCT02915445), breast cancer (NCT02915445), stomach neoplasms (NCT02725125), liver neoplasms (NCT02729493) and small cell lung cancer (NCT03392064).

**TABLE 3:** Registered clinical trials of CSCs‐targeted CAR‐T therapy

Target	Tumour target	Sponsor	NCT number	Phase	Status	Start date
EpCAM	EpCAM‐positive cancer	First Affiliated Hospital of Chengdu Medical College	NCT03013712	Phase I/II	Recruiting/unknown	2017
	Nasopharyngeal carcinoma or breast cancer	Sichuan University	NCT0avb2915445	Phase I	Recruiting	2016
	Advanced gastric cancer with peritoneal metastasis	West China Hospital, Sichuan University	NCT03563326	Phase I	Recruiting	2018
	Advanced solid tumour neoplasms	Tang‐Du Hospital	NCT04151186	NA	Not yet recruiting	2019
	Stomach neoplasms	Sinobioway Cell Therapy Co., Ltd.	NCT02725125	Phase II	Unknown	2015
	Liver neoplasms	Sinobioway Cell Therapy Co., Ltd.	NCT02729493	Phase II	Unknown	2015
CD133	Relapsed and/or chemotherapy refractory advanced malignancies	Chinese PLA General Hospital	NCT02541370	Phase I/II	Completed	2015
	Relapsed/refractory acute myeloid leukaemia	Zhujiang Hospital	NCT03473457	NA	Recruiting	2018
CD123	Acute myeloid leukaemia (AML)	Hebei Senlang Biotechnology Inc., Ltd.	NCT03796390	Phase I	Recruiting	2018
	AML	University of Pennsylvania	NCT03766126	Phase I	Active, not recruiting	2018
	CD123+ AML	Beijing Immunochina Medical Science & Technology Co., Ltd.	NCT03585517	Phase I	Recruiting/unknown	2018
	Relapsed/refractory AML	Cellectis S.A.	NCT03190278	Phase I	Recruiting	2017
	Relapsed/refractory AML	The Affiliated Hospital of the Chinese Academy of Military Medical Sciences	NCT03556982	Phase I/II	Recruiting/unknown	2018
	Relapsed/refractory AML	Chongqing Precision Biotech Co., Ltd	NCT04265963/NCT04272125	Phase I/II	Recruiting	2020
	Relapsed/refractory AML	Zhujiang Hospital	NCT03473457	NA	Recruiting	2018
	Relapsed/refractory AML	Shenzhen Geno‐Immune Medical Institute	NCT03222674	Phase I/II	Unknown	2017
	CD123+ acute myeloid leukaemia	Wuhan Union Hospital, China	NCT04014881	Phase I	Recruiting	2019
	Acute myeloid leukaemia or blastic plasmacytoid dendritic cell neoplasm	City of Hope Medical Center	NCT02159495	Phase I	Recruiting	2015
	Myeloid malignancies	Southwest Hospital, China	NCT02937103	Phase I/II	Recruiting	2016
	Adult acute myeloid leukaemia	Affiliated Hospital to Academy of Military Medical Sciences	NCT03114670	Phase I	Recruiting	2017
	B‐cell malignancies	Shenzhen Geno‐Immune Medical Institute	NCT03125577	Phase I/II	Recruiting	2019
	B‐cell leukaemia	Shenzhen Geno‐Immune Medical Institute	NCT04016129	Phase I/II	Recruiting	2019
	CD19‐Negative B‐cell malignancies	Shenzhen Geno‐Immune Medical Institute	NCT04430530	Phase I/II	Recruiting	2020
	AML	St. Jude Children's Research Hospital	NCT04318678	Phase I	Recruiting	2020
	Refractory/relapsed acute leukaemia	Second Affiliated Hospital of Xi'an Jiaotong University	NCT03672851	Phase I	Terminated (adverse effect)	2019
	paediatric subjects with relapsed/refractory AML	University of Pennsylvania	NCT04678336	Phase I	Recruiting	2020
	Relapsed/refractory AML	Fujian Medical University	NCT03631576	Phase II/III	Recruiting	2018
DLL3	Small cell lung cancer (SCLC)	Amgen	NCT03392064	Phase I	Suspended	2018
CD44v6	AML or multiple myeloma (MM)	AGC Biologics S.p.A.	NCT04097301	Phase I/II	recruiting	2019
	Cancers which are CD44v6 positive	Shenzhen Geno‐Immune Medical Institute	NCT04427449	Phase I/II	Recruiting	2020
	Breast cancer	Shenzhen Geno‐Immune Medical Institute	NCT04430595	Phase I/II	Recruiting	2020

Among the clinical trials, a CD133‐targeted Phase I/II clinical study at Chinese PLA General Hospital for treating the relapsed and/or chemotherapy refractory advanced malignancies is the world's first successful clinical trial of CSCs‐targeted CAR‐T therapy for cancer treatment (NCT02541370).^57^ CD133 is a marker of CSCs and endothelial progenitor cells (EPCs) which had been scientifically proven to be involving in tumour metastasis and recurrence.^58^ In this clinical trial, 23 patients were enrolled, 14 of which are with hepatocellular carcinoma [HCC], seven of which pancreatic carcinomas and two of which colorectal carcinomas. Finally, three subjects achieved partial remission (PR) and 14 subjects' condition became stable with no serious adverse events. However, a side effect, namely, “on‐target, off‐tumour” effects, manifested as haematopoietic system toxicities were observed in most subjects in the study. This might be caused by the expression of CD133 on the surface of CD34+ progenitor cells in adult bone marrow and peripheral blood.^59^ Moreover, cells with expressed non‐targeted antigen might give rise to tumour recurrences though CD133‐targeted CAR‐T cells eliminate target‐express cells. The process indicates antigen escape. Therefore, avoiding rapid growth of antigen‐negative cells after the antigen‐positive cell clearance is of great importance for improving clinical application of CSCs‐targeted CAR‐T therapy.

Side effects in patients treated with CSCs surface antigen‐targeted CAR‐T therapy were also shown in several literature. In a pilot trial for studying the safety of anti‐CD123 CAR‐T cell product, the fourth‐generation, apoptosis‐inducible lentiviral CAR‐T cells targeting CD123 (4SCAR123 T cells) were used to treat a patient with AML‐M2,^60^ who was administered with cyclophosphamide (CTX) as conditioning regimen for 3 consecutive days and was subsequently infused with 1.8 × 10^6^/kg anti‐CAR123 T cell. One day after the infusion, the patient had rigorous chills and fevers, low blood pressure and hypoxaemia. The subject also suffered from severe cytokine release syndrome (CRS) 4 days after the infusion, because of the controlling effects from administration of one dose tocilizumab. Fortunately, in this first human experiment of CD123‐specific CAR‐T cells for AML treatment, obvious off‐target cytotoxicity from the 4SCAR123 T cells were not found except for a decrease of blasts 20 days after CAR‐T therapy.^60^ Apart from that, one side effect caused by CSCs‐targeted CAR‐T therapy was shown in a clinical trial registered in ClinicalTrials.gov (NCT03672851). The trial was a Phase I study designed to determine the safety and efficacy of anti‐CD123 CAR‐T cells in treating patients diagnosed with refractory/relapsed acute leukaemia and conducted in a dose‐escalation administration pattern. Unfortunately, it was terminated due to side effects while the experiment results was not released. Based on the experimental design, only two subjects were enrolled. The situation might be caused by individual differences of the included cases or the unreasonable design of the CAR structure. With limited subjects, the study was ill‐grounded in proving that CD123 was unsuitable for CAR‐T cell targeting.

## 6 CHALLENGES AND FUTURE DIRECTIONS

Although some successful animal experiments conducted with CSCs‐targeted CAR‐T cells have been reported and some ongoing CSCs‐targeted CAR‐T therapy clinical trials have shown good tumour treatment prospects, many challenges in clinical application exist. (Table 4).

**TABLE 4:** Challenges and overcoming strategies of CSCs‐targeted CAR‐T therapy

Challenges	Overcoming strategies
Toxicity	Designing and using dual‐targeted CAR‐T cells^64^ Intratumoural injection of CAR‐T cells^65^ Safety switches: inactivate CAR‐T cells through the transduction of so‐called suicide genes in the event of severe toxicity^66^ Using CAR‐T cells expressing an inhibitory chimeric antigen receptor^21^ The administration of tocilizumab or steroid therapy enables improved control of cytokine release syndrome(CRS)^68^, ^69^ Using corticosteroids, antiepileptics and care measures with intensive care unit monitoring for the management of immune effector cell‐associated neurotoxicity syndrome (ICANS)^12^
Persistence and potency	Costimulatory signalling domain optimization: incorporate one or more costimulatory signal domains, like 4‐1BB, OX40, CD27 or ICOS^72^, ^73^, ^74^ Combination therapy between CAR‐T cells and immune checkpoints inhibitors, such as anti‐PD‐1, anti‐CTLA‐4, anti‐TIM3, anti‐LAG3 and anti‐adenosine 2A receptor (A2AR)^74^ Transgenic cytokine expression of IL‐12, IL‐18, IL‐7, IL‐15 and IL‐21 cytokines on CAR‐T cells^78^ Construct CAR‐T cells capable of inducing JAK/STAT signalling upon antigen stimulation^79^
Trafficking	The local infusion of CAR‐T cells to tumour sites^81^ Modulation of chemokine signalling: overexpressing CCR4,CCR2b or CXCR3 ligands on CSCs‐targeted CAR‐T cells^82^, ^83^, ^84^ First using traditional therapies to remove most of the tumour cells, and then applying CAR‐T therapy to target CSCs^85^
Infiltration	Targeting tumour stromal cells with fibroblast activation protein (FAP)‐directed CAR‐T cells to inhibit matrix production and angiogenesis^87^ Directing CAR‐T cell to target vascular endothelial growth factor receptor 2(VEGFR2)^88^ Engineering CAR‐T cells to secrete extracellular matrix (ECM)‐modifying enzymes heparanase^89^
Immunosuppressive tumour microenvironment	Constructing CSCs‐targeted CAR‐T cells overexpressing IL‐12, IL‐18, IL‐7, IL‐15 and IL‐21 cytokines to provide cytokine support in the immunosuppressive tumour microenvironment(TME)^78^ Neutralization of immunosuppressive mediators within the TME, such as TGF‐β, IL‐10 and arginase I^92^ The combination with checkpoint inhibitors: PD‐1/PD‐L1 or CTLA‐4 blocking antibodies^96^, ^97^
Heterogeneity	Engineering bispecific CAR‐T cells by designing a single CAR molecule with two (or more) distinct binding domains^103^ Multi‐target CAR‐T cell therapies: creation by mixing different CAR‐T cell products targeting single antigens prior to infusion, or transducing T cells with multiple CAR constructs^13^ CAR‐T cells expressing bispecific T‐cell engagers (BiTEs) to recruit bystander T cells against a second tumour‐associated surface antigen^104^, ^105^, ^106^

### 6.1 Toxicity

One of the limitations in CAR‐T therapy is on‐target off‐tumour toxicity, caused by the direct attack on normal cells which have the shared expression of the targeted antigen.^61^ On‐target/off‐tumour toxicity becomes a major hindrance of CSCs‐targeted CAR‐T therapy, because in normal cells, some CSCs markers are found, such as CD133 expressed in normal neural stem cells or ALDH expressed in hematopoietic stem cells.^62^, ^63^ To reduce the toxicity, selecting a safer antigen of CAR‐T cells is needed or designing dual‐targeted CARs to enhance the tumour specificity of CAR‐T cells may work.^64^ Toxicity can also be minimized by local (intratumoural) delivery of CSCs‐targeted CAR‐T cells.^65^ Moreover, introducing suicidal genes as a “safety switch” in CAR‐T cells when adverse reactions are uncontrollable may limit on‐target, off‐tumour toxicities.^66^ Similarly, modifying CAR‐T cells and enabling them to express an inhibitory chimeric antigen receptor, such as CTLA‐4 or PD‐1, can achieve antigen‐specific suppression of T‐cell cytotoxicity, cytokine release and proliferation.^21^

Adverse events other than on‐target/off‐tumour toxicity include cytokine release syndrome (CRS) and immune effector cell‐associated neurotoxicity syndrome (ICANS), which were common toxicities of CAR‐T cells in treating tumours.^67^ In the first several days after CAR‐T cell infusion, CRS is mostly found and patients may have fever, hypotension and tachycardia which might lead to haemodynamic instability, causing end‐organ injury; after the onset of CRS, neurotoxicity syndrome, which manifests as subtle cognitive decline, may occur.^12^ CRS is associated with elevated IL‐6 levels in patients receiving CAR‐T therapy and anti‐IL‐6 receptor antagonist tocilizumab is, thus, used to treat CRS. For instance, FDA sanctified the use of the drug in the treatment of CRS when the first CAR‐T cell product was approved.^68^ Corticosteroids, which suppress immune responses, are also commonly used in the management of the toxicity once the patient does not have a rapid response to IL‐6 receptor blockade.^69^ Alternatively, therapeutic options for ICANS are corticosteroids, antiepileptics and care measures with intensive care unit (ICU) monitoring.^12^

### 6.2 Persistence and potency

A large number of clinical trials of CAR‐T cell therapy show the poor persistence of infused T cells, especially in solid tumours.^70^ Therefore, improving the persistence and efficacy of CAR‐T cells has become one of the focuses in current research on CSCs‐targeted CAR‐T therapy. CAR structure and T cells exhaustion determine the cells persistence, which can be enhanced through improving the costimulatory domain of CAR‐T cells.^71^ CAR‐T cell proliferation, persistence and potency can be elevated when one or more costimulatory signal domains were incorporated, such as 4‐1BB, ICOS, OX40 or CD27, which has been made clear in preclinical studies.^72^, ^73^, ^74^ Moreover, several studies have proven that CAR‐T cell persistence was better maintained through incorporation of immune checkpoint blockade into CAR‐T cells, and such immune checkpoint inhibitors include anti‐programmed cell death protein 1 (PD‐1), anti‐cytotoxic T‐lymphocyte antigen‐4 (CTLA‐4), anti‐mucin domain‐containing protein 3 (TIM3), anti‐Lymphocyte activation gene 3 (LAG3) and anti‐adenosine 2A receptor (A2AR).^74^ In addition, optimizing T‐cell activation, expansion and persistence require not only antigen participation (signal1) or costimulatory signals (signal2), but also cytokine support (signal3).^75^ Providing cytokine signals promotes the activation and proliferation of CAR‐T cells. However, some clinical trials found adverse reactions when patients were directly administered with exogenous cytokines.^76^, ^77^ In order to minimize systemic toxicity and induce the accumulation of high cytokine concentrations at the tumour site, CAR‐T cells were modified to produce IL‐12, IL‐18, IL‐7, IL‐15 and IL‐21 cytokines, and activation and persistence of CAR‐T cells were, therefore, enhanced *in vivo*.^78^ Similar to the forced expression of cytokine genes in CAR‐T cells, constructing CAR‐T cells with the capability of inducing cytokine signalling upon antigen stimulation can also provide cytokine support. Based upon that, a novel CAR containing a JAK‐STAT signalling domain has been developed by Kagoya et al.^79^ This invention incorporated cytoplasmic domain of IL‐2 receptor β between the cytoplasmic domains of CD28 and CD3z for JAK‐STAT pathway activation and a YXXQ motif at the C‐terminus of CD3z for STAT3 recruitment. The novel CAR demonstrated superior *in vivo* persistence and antitumour effects in models of liquid and solid tumours. All the approaches mentioned above to improve the persistence and potency of CAR‐T cells are good references in CSCs‐targeted therapy.

### 6.3 Trafficking

Apart from effectively treating haematological tumours, the CSCs‐targeted CAR‐T therapy is applicable to kill cancer stem cells in solid tumours in radical treatment. A major challenge in solid tumour treatment is less trafficking of CAR‐T cells into these sites, since CSCs in solid tumours are less possible to access immune cells and are usually surrounded by compact stroma and tumour cells.^80^ We proposed the following methods currently used to improve the trafficking or homing ability of CAR‐T cells so as to overcome the challenge. The most straightforward method is to directly infuse the CSCs‐targeted CAR‐T cells into tumour sites. In a previous report, the local infusion of CAR‐T cells resulted in significant regression of glioblastoma. Nevertheless, localized therapy is not suitable for many metastatic solid tumours.^81^ Modulation of chemokine signalling would be another choice to elevate the trafficking ability of CSCs, as numerous chemokines mediate immune cell trafficking. Several preclinical models demonstrated that the forced expression of a chemokine receptor of CCR4, CCR2b or CXCR2 improved the homing ability of CAR‐T cells.^82^, ^83^, ^84^ Since CSCs are encompassed by a bulk of tumour cells, we proposed that combining CSCs‐specific CAR‐T and conventional anticancer therapies may be effective. Specifically, it is recommended that traditional therapies, including radiotherapy or chemotherapy are used to remove most of the tumour cells for better exposure of CSCs, and CSCs‐targeted CAR‐T cells are subsequently infused for full eradication of the tumour.^85^

### 6.4 Infiltration

After CAR‐T cells reach the tumour site, an issue needs to be considered how the cells should approach CSCs expressing target antigens to form immune synapses and destroy tumour stem cells. Due to the physical and biochemical barriers established by the extracellular matrix (ECM) around CSCs, the infiltration of CAR‐T cells has become one challenge.^80^, ^86^ Fibroblast activation protein (FAP) is a surface marker of cancer‐associated stromal cells (CASCs), and has a role in remodelling ECM. FAP‐targeted CAR‐T cells have the ability to break through the physical barrier established by ECM, which is achieved by targeting PAF+CASCs to inhibit matrix production and angiogenesis.^87^ Directing CAR‐T cell to target vascular endothelial growth factor receptor 2(VEGFR2) is another way to improve penetration and antitumor response, which is achieved by destroying tumour vascular endothelial cells.^88^ In addition, Caruana et al. reported that engineering CAR‐T cells to express ECM‐degrading enzyme heparanase improved the infiltration of T cells in tumours.^89^

### 6.5 Immunosuppressive tumour microenvironment

Cancer stem cells survive in an immunosuppressive tumour microenvironment (TME) composed of vascular niches, cancer‐associated fibroblasts, cancer‐associated mesenchymal stem cells, hypoxia, tumour‐associated macrophages and extracellular matrix, which hinders the direct killing of tumour stem cells by one's own immune cells and the adoptive CAR‐T cells.^90^ Therefore, combining CSCs‐targeted CAR‐T therapy and the strategy of targeting the immunosuppressive TME of CSCs may help improve the efficiency of CSCs removal. As we mentioned in “persistence and potency” section in this review, cytokine support served as one of the important signals for optimal T‐cell activation and proliferation. However, this signal was lacking in the TME of CSCs.^91^ Constructing CSCs‐targeted CAR‐T cells which overexpress IL‐12, IL‐18, IL‐7, IL‐15 and IL‐21 cytokines may be an effective way to provide support for the activation, proliferation and killing of CSCs of CAR‐T cells in immunosuppressive TME.^78^

Several studies have proved that CSCs have the ability to evade the immune system, because these cells secrete several substances into the TME, such as TGFβ, IL‐10, IL‐4 and IL‐13, which exert inhibitory effects on an array of immune cells.^3^ Neutralization of immunosuppressive mediators within the TME is another way to enhance the potency of CSCs‐targeted cells. For example, Takahashi has proved that the neutralization of TGF‐β, IL‐10 and arginase I with anti‐TGF‐β mAb, anti‐IL‐10 mAb and the arginase I inhibitor Noha, or L‐arginine significantly restored T‐cell proliferation.^92^

Several other studies showed that the immunosuppressive TME can be triggered by CSCs through the mechanism of up‐regulated expression of PD‐L1 on the CSCs' surfaces.^93^, ^94^ PD‐L1 suppresses CAR‐T cells' functions and induces their exhaustion upon binding to PD‐1 on activated T cells.^95^ Therefore, combining CSCs‐targeted therapy with FDA‐approved PD‐1/PD‐L1 checkpoint inhibitors, including three for PD‐1 (pembrolizumab, nivolumab and cemiplimab) and three for PD‐L1 (atezolizumab, avelumab and durvalumab), may be a choice to mitigate the immunosuppressive microenvironment.^96^ Fang Zheng et al. have observed significant antitumor effects and dramatic ALDH^high^ CSCs elimination, following the triple therapy of the dual blockade of PD‐L1 and CTLA‐4 and CSC‐DC vaccine which were accompanied by significantly enhanced T‐cell expansion, suppressed TGF‐β secretion, enhanced IFN‐γ secretion and significantly enhanced host‐specific CD8^+^ T cell response against CSCs.^97^ Based upon all the results from the literature, administration of a‐PD‐L1 and a‐CTLA‐4 blockade combined with CSCs‐targeted CAR‐T cells may be an effective immunotherapeutic strategy for cancer patients.

### 6.6 Heterogeneity

One great challenge in cancer therapy is intratumour heterogeneity, while CSCs are one of the determining factors causing the problem.^98^ Therefore, eradication of CSCs by CAR‐T therapy is promising for overcoming the heterogeneity. However, accumulating evidence suggests that CSCs represent phenotypically and functionally heterogeneous populations, which has been found in colorectal,^99^ colon,^100^ hepatocellular^101^ and breast cancer stem cells,^102^ leading to antigen loss or escape when applying CSCs‐targeted CAR‐T therapy.

Given that CD19/CD22 bispecific CAR‐T cells have demonstrated clinical efficacy in patients with B‐cell malignancies,^103^ bispecific CAR‐T cells can be bio‐engineered by designing a single CAR molecule with two (or more) distinct binding domains of CSCs‐specific markers, so as to overcome antigen escape caused by CSCs heterogeneity. Furthermore, multi‐target CAR‐T cell therapies can be created to overcome the limitation of antigen loss by mixing different CAR‐T cell products targeting single antigens prior to infusion or by transducing T cells with multiple CAR constructs.^13^

Transgenic modification of the CAR structure to elicit an endogenous immune response through recruiting additional effector cells is an alternative approach to avoid the heterogeneity. To recruit bystander T cells against a second tumour‐associated surface antigen, CAR‐T cell targeting can be combined with the release of bispecific T‐cell engagers (BiTEs).^104^ Iwahori et al. first reported the generation of T cells which can secrete a bispecific T‐cell engager specific both for CD3 and the tumour‐associated antigen, erythropoietin‐producing hepatocellular carcinoma A2 (EphA2), for bystander T‐cell‐mediated *in vitro* cytolysis.^105^ More recently, EGFRvIII‐targeted CAR‐T cells were constructed to secrete engagers against wild‐type EGFR for local recruitment of bystander T cells against EGFRvIII‐negative tumour cell subpopulations in glioblastoma, so as to overcome the limitation of antigen escape.^106^

## 7 CONCLUSION

Due to CSC's characteristics of self‐renewal, multi‐lineage differentiation, tumour‐formation ability and chemo‐radio‐resistance, the CSCs existence is the key factor causing cancer treatment failure. The advantages of CAR‐T therapies are recognizing specific surface antigen, activating T cells in an MHC‐unrestricted manner, long‐term *in vivo* persistence and proliferation, and efficient homing of CAR‐T cells to the tumour site, which are believed to be a potential method to eliminate CSCs. Apart from the detailed analysis of the feasibility and advantages of targeting CSCs with the bio‐modified CAR‐T cells, the paper also reviewed the few existing reported laboratory results and summarized the registered clinical trials about this method. Finally, we discussed the current challenges of this therapy and the solutions that can be adopted for the future development of CSCs‐targeted therapy. As the first therapy mentioned in the review has the potential to cure cancers and is currently on the market, harnessing CAR‐T cells to target CSCs is believed to achieve greater success in treating tumours.

## CONFLICT OF INTEREST

Conflict of interest relevant to this article was not reported.

## AUTHOR CONTRIBUTIONS

**Xiaoyue Cui:** Conceptualization (lead); Data curation (lead); Formal analysis (lead); Investigation (lead); Resources (lead); Software (lead); Writing‐original draft (lead); Writing‐review & editing (lead). **Rui Liu:** Data curation (equal); Supervision (supporting); Writing‐review & editing (equal). **Lian Duan:** Investigation (equal); Writing‐review & editing (supporting). **Dan Cao:** Data curation (supporting); Investigation (supporting). **Qiaoling Zhang:** Data curation (supporting); Writing‐review & editing (supporting). **Aijie Zhang:** Resources (equal); Writing‐review & editing (equal).
